# Cryptic Diversity and Genetic Variation in a Korean Endemic Freshwater Fish, the Korean Dark Chub (*Zacco koreanus*) Compared With Its Relative *Zacco temminckii*

**DOI:** 10.1002/ece3.73451

**Published:** 2026-04-08

**Authors:** Yu Rim Kim, Ji Eun Jang, Hee‐kyu Choi, Soon Young Hwang, Ji Young Kim, Soonku So, Hyuk Je Lee

**Affiliations:** ^1^ Molecular Ecology and Evolution Laboratory, Department of Biological Science Sangji University Wonju Republic of Korea; ^2^ National Park Research Institute, Korea National Park Service Wonju Republic of Korea

**Keywords:** cryptic species, endemic species, genus *Zacco*, phylogeography, speciation, species complex

## Abstract

Species boundaries are not always straightforward, especially during early stages of divergence, when gene flow may still take place. The Korean dark chub *Zacco koreanus*, a widely distributed freshwater fish endemic to Korea, was recently classified as a species distinct from its congener *Z. temminckii* . However, morphological differences between the two species are ambiguous and genetic differences between and within species remain largely unexplored. In this study, using mitochondrial DNA cytochrome oxidase I (COI), control region (CR) and nine newly developed microsatellite loci, we determined phylogenetic relationships and population genetic structure of *Z. koreanus* among six geographically separated river basins in comparison with *Z. temminckii* . We found that *Z. koreanus* comprises relatively well‐separated mitochondrial clades associated with major river basins, and that some individuals morphologically identified as *Z. koreanus* were genetically assigned to *Z. temminckii* , suggesting the possibility of natural hybridization between the two species. *Z. koreanus* individuals from the Nakdong and Seomjin Rivers are the most closely related to *Z. temminckii* , and they probably represent cryptic species, as they showed a genetic distance of 2.2%~2.9%, although *Z. koreanus* had a distinct genetic structure. Comparisons of the whole mitogenomes from different rivers further suggest that the Nakdong and Seomjin River populations may represent divergent evolutionary lineages within *Z. koreanus* and possible cryptic diversity. The results of this study provide insight into understanding how Korean freshwater fishes have evolved in response to geographically isolated major rivers. The evolutionary mechanisms underpinning the cryptic species diversity in freshwater systems are worthy of further study.

## Graphical abstract

The phylogenetic relationship analyses revealed four distinct lineages within *Zacco koreanus*, with the Nakdong and Seomjin River populations showing approximately 2.6% genetic divergence from other lineages—comparable to the interspecific distance between *Z. koreanus* and *Z. temminckii* (~2.7%). These results indicate ambiguous species boundaries and potential cryptic speciation within *Z. koreanus*, suggesting that *Z. koreanus* and *Z. temminckii* together may constitute a species complex, thereby warranting a re‐evaluation of their taxonomic status through integrative genomic and ecological approaches.

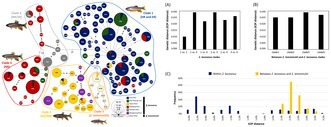

## 1 ׀ Introduction

Speciation is the process by which preexisting populations or species evolve into new and distinct species through continual formation of independently evolving lineages (Wolf et al. 2010; Zozaya et al. 2023). Speciation modes include allopatric speciation, in which speciation occurs with limited gene flow due to physical isolation by geographic barriers, and sympatric speciation, where it arises from ecologically‐based reproductive isolation in the absence of physical barriers (Mayr 1963; Coyne and Orr 2004; Pyron and Burbrink 2010). The mechanisms underlying speciation have long been studied, but the topic still remains an open question (Sobel et al. 2010; Matute and Cooper 2021). Studying speciation– the process by which new species arise– is essential for understanding how biodiversity evolves (Network, T. M. C. S 2012).

Ecological differentiation is an evolutionary driving force that promotes not only interpopulation divergence, but also even speciation through various mechanisms (Schluter 2000; Rundle and Nosil 2005; Gorospe et al. 2025). Ecological speciation occurs when adaptation to different environments leads to reproductive isolation (Rundle and Nosil 2005). This process can arise through habitat isolation without geological barriers, temporal differences in reproduction, and/or divergence in mating signals, all of which can reduce opportunities for interbreeding (Rice 1987; Sobel et al. 2010; Servedio and Boughman 2017). Collectively, these mechanisms illustrate how ecological differentiation can generate barriers to gene flow and promote the formation of new species (Rundle and Nosil 2005; Nosil 2012). Adaptive ecological divergence among populations by natural selection in response to different habitat environments can occur even in a short period of time (Le Cam et al. 2015; Munar‐Delgado et al. 2024). Elucidating the mechanisms of ecological divergence by which geographically isolated populations speciate can address the key question in a field of evolutionary ecology. Nevertheless, species boundaries are not always clear‐cut, especially during early stages of divergence when gene flow may still occur among lineages (Leliaert et al. 2014). Moreover, species delimitation may be challenging because of incomplete lineage sorting, introgression, cryptic species diversity, or phenotypic diversification within species (Leliaert et al. 2014; Huang 2020; Kang et al. 2020; Jang et al. 2021; Kabus et al. 2025).

Phylogeographic investigations can help understand the evolutionary mechanisms and processes underlying reproductive isolation among natural populations (Beheregaray 2008) and also allow testing biogeographic hypotheses more specifically (Won et al. 2020; Taniguchi et al. 2021). Moreover, phylogeographic studies enable disentangling the evolutionary processes shaping the current population genetic structure of species and also elucidate the historical/demographic events, which might have caused speciation events in the past (Fratini et al. 2005; Avise 2012). In particular, phylogeographic analysis has been proven powerful to study the pattern and process of allopatric speciation in freshwater fishes with regard to their geographical distribution patterns and phylogenetic relationships among populations. For example, eight river populations of Chinese hook snout carp ( *Opsariichthys bidens* ) showed a mean genetic distance of 16.8% among populations based on mtDNA cyt *b* strongly suggests the possibility of cryptic or distinct species (Li et al. 2009). According to Jeon et al. (2020), a spined loach *Cobitis nalbanti*, which occurs in separate river basins on the Korean Peninsula, have expanded their distributional range into northern rivers via the paleo‐Yellow River system during glacial periods. *Cobitis nalbanti* from the Tamjin River basin in southern parts of South Korea had close phylogenetic relationships with Japanese *Cobitis*, suggesting that there was a land bridge occurring at times of lower sea level—a freshwater passage between the Korean Peninsula and Japanese archipelago in the past. During the Pleistocene, sea‐level fluctuations repeatedly altered the geography of East Asia. When sea levels dropped by approximately 150~160 m, land bridges across the Tsushima Strait intermittently connected between the Korean Peninsula and the Japanese Archipelago (Lee et al. 2008). These connections likely occurred around 1.2, 0.63, and 0.43 Ma and likely facilitated dispersal of East Asian organisms, including freshwater fishes (Aoki et al. 2021; Taniguchi et al. 2021). In this regard, phylogeographic studies allow clarification of the genetic characteristics and phylogenetic relationships across populations spanning different river basins, particularly for species with a wide distributional range. Consequently, it is important to study phylogenetic differences among populations according to geographic distribution patterns, in order to understand how different clades evolve in different geographic settings in freshwater fishes in Korea (Jang et al. 2017; Jeon et al. 2020; Choi and Lee 2025).

The formation of major river basins in the Korean Peninsula, including the Han, Nakdong, Geum, and East‐flowing rivers was influenced by the interplay between geological and glacial activities during geological history (NIBR 2011; Lee et al. 2024). Geographic isolation by physical barriers among natural populations may facilitate genetic differentiation or divergence via limited gene flow among the river basins, which can lead to ecotype formation and even speciation through adaptations to different environmental conditions by divergent natural selection (Kim et al. 2003; Coyne and Orr 2004; Jeon et al. 2022). Geographically separated populations of freshwater fishes among different river drainages in Korea have evolved a number of endemic species, such as a spined loach (*Iksookimia pacifica*), a torrent catfish ( *Liobagrus mediadiposalis* ), a Korean splendid dace ( *Coreoleuciscus splendidus* ), a Korean dark chub (*Zacco koreanus*), and other species (Kim et al. 2003). There are 213 freshwater fishes identified in South Korea, of which 67 (approximately 32%) are recognized as Korean endemic species (NIBR 2013).

Mitochondrial DNA (mtDNA) has been widely used as a molecular marker for phylogeographic studies (Avise 2012). Because of its maternal inheritance, high mutation rate, and mutations being inherited from maternal ancestors to descendants without recombination, mtDNA is useful for studying genetic diversity, structure, evolutionary (phylogeographic) history, and species identification (Brown et al. 1979; Galtier et al. 2009). However, mtDNA also has some limitations, as it represents a single maternally inherited locus and may not fully reflect the evolutionary history of species due to processes such as incomplete lineage sorting, introgression, or mito–nuclear discordance (Funk and Omland 2003; Toews and Brelsford 2012). Several mtDNA‐based phylogeographic studies have been conducted on the Korean freshwater fishes, and they found that distinct lineages tend to evolve in geographically disconnected, distinct river basins (Jang et al. 2017; Baek et al. 2018; Jeon et al. 2018; Kim et al. 2023; Choi and Lee 2025). However, a majority of such studies have primarily focused on endangered species with relatively limited geographic distributions, which presents a caveat in terms of comparing genetic structure between river basins spanning large areas of the Korean Peninsula.

**FIGURE 1:**
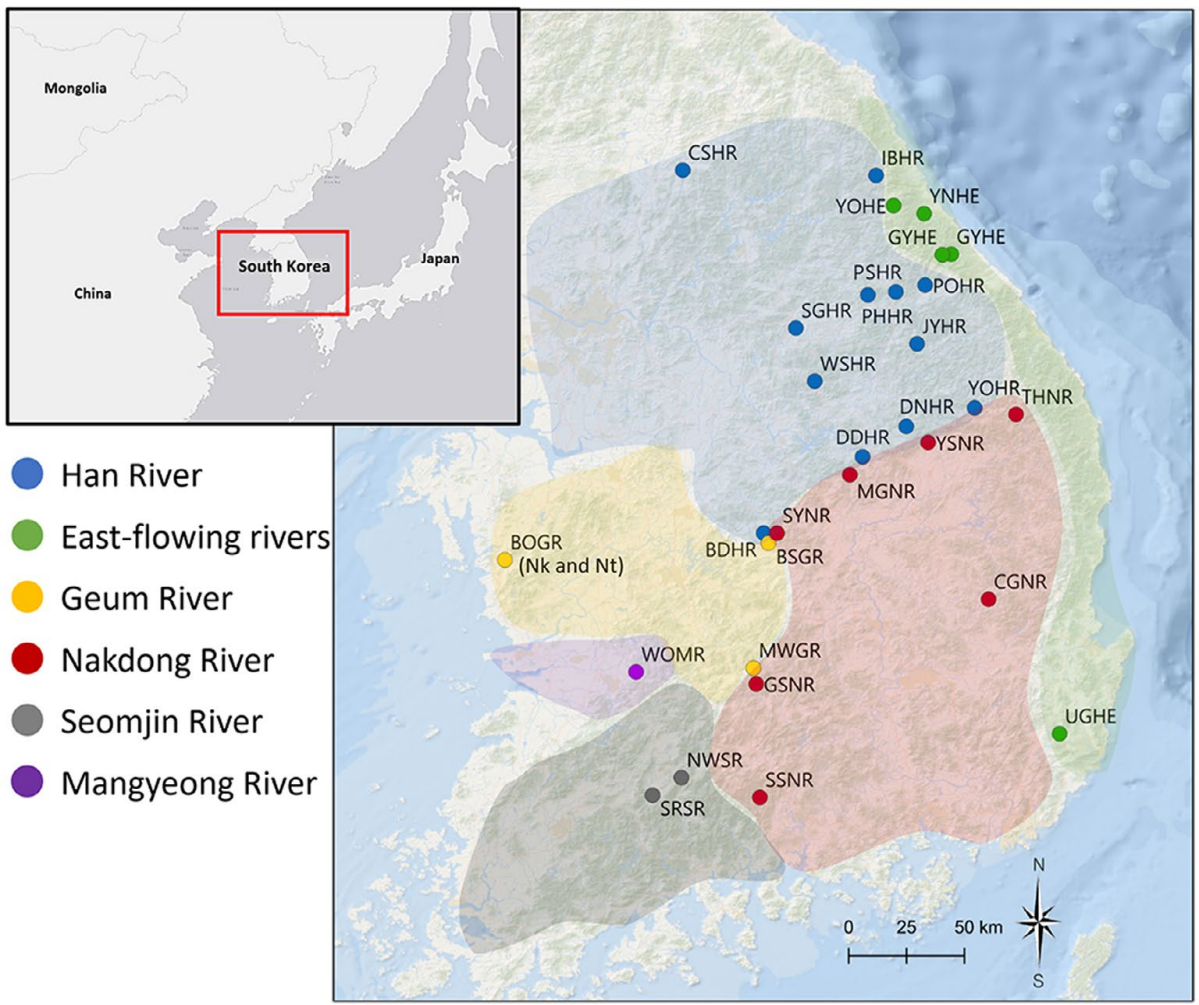
A sampling localities for *Zacco koreanus* and *Z. temminckii* in South Korea. *Z. temminckii* was collected only from BOGR (Zt). Circles in different colors denote sampling locations in different river basins.

The Korean dark chub *Zacco koreanus* (*Nipponocypris*; family Xenocyprididae) is widely distributed in most Korean rivers/streams, such as the Han, Geum, Seomjin, and Nakdong rivers (Figure 1). This fish has been commonly used for evaluating stream environmental conditions because of its high number of individuals and broad distributional range (Kim et al. 2005; Lee et al. 2013). This species was previously considered as *Zacco temminckii* (*Nipponocyris temminckii*), but has been classified as a separate species since 2005, based on its geographic distribution, morphological characters, and genetic differences (Kim et al. 2005). However, morphological differences between the putative two species are often ambiguous, and levels of genetic and morphological differences between/within species also remain unclear (Kim 2021). In addition, it has been suggested that *Z. koreanus* has evolved three ecotypes with respect to body shape and coloration in association with its geographical distribution (Chae and Yoon 2006). Yet, there have been no studies to date that have investigated quantitatively genetic differentiation, morphological differences, and phylogenetic relationships between these ecotypes and the species.

In this study, we investigated the phylogeographic relationships and population genetic structure among 28 populations of *Z. koreanus* and one *Z. temminckii* population using mtDNA [cytochrome oxidase I (COI) and control region (CR)] and nine newly developed microsatellite loci. Our study had three specific objectives, which were: (1) to understand the phylogenetic relationships among populations of *Z. koreanus* (along with *Z. temminckii* ) from six geographically separated river basins (the Han, East‐flowing, Geum, Nakdong, Seomjin, and Mangyeong rivers) across nearly its entire distributional ranges; (2) to determine the population genetic structure of *Z. koreanus* among/within the river basins; (3) to verify genetically the species boundaries between *Z. koreanus* and *Z. temminckii* and evaluate the possibility of a species complex. The results of our study provide insight into how Korean freshwater fishes evolved in response to geographically isolated major river/stream environments in general.

## 2 Materials and Methods

### 2.1 Study Sites and Sample Collection

From April 2020 to August 2023, we collected a total of 829 individuals of *Z. koreanus* [mtDNA (*N* = 754), microsatellites (*N* = 717)] from 28 different localities within five river basins [Han River (HR; number of populations, *N* = 12), East‐flowing rivers (ER; *N* = 4), Geum River (GR; *N* = 3), Nakdong River (NR; *N* = 7), Seomjin River (SR; *N* = 1) and Mangyeong River (MR; *N* = 1); Figure 1, Table 1] in South Korea using dip nets (5 × 5 mm mesh size) and cast nets (7 × 7 mm mesh size). Thirty individuals of *Z. temminckii* [mtDNA (*N* = 23), microsatellites (*N* = 30)] were sampled at one site from the Geum River. The collected *Z. koreanus* and *Z. temminckii* individuals were individually photographed in the field. The tip of the caudal fin, which can be regenerated, was collected, and the fish were then immediately released back to the original sites (Fu et al. 2013). The collected caudal fins were preserved immediately in a microcentrifuge tube containing 99.9% ethanol and refrigerated at 4°C. The sampling was carried out as part of research projects of the National Research Foundation of Korea (NRF) (RS‐2024‐00412091) and the Korea National Park Research Institute (NPRI 2022‐05). All experimental procedures involving fish were conducted in accordance with institutional guidelines for the care and use of animals and were approved by the Institutional Animal Care and Use Committee of Sangji University.

**TABLE 1:** Summary of genetic diversity statistics in 29 populations (including 28 populations of *Zacco koreanus* and one population of *Z. temminckii* ) from six river basins at mitochondrial DNA (COI and CR; 1,457 bp) and nine microsatellite loci. Three populations (WSHR, YNHE, CGNR) with insufficient sample sizes (*N* < 5) were excluded from some of the genetic diversity analyses (e.g., *Hr*, *H*, *π*, AR, *H*_E_, *H*_O_, *F*_IS_ and HWE).

River basin	Pop ID	Coordinates	MtDNA						Microsatellites								
			*N*	*N*_H_	*Hr*	PH	*H*	*π*	*N*	*N*_A_	AR	PA	*H*_E_	*H*_O_	*F*_IS_	*N*_E_	HWE
Han River	IBHR	38°13′06.87″ N 128°22′15.30″ E	30	13	6.55	4	0.90	0.002	30	11.8	8.6	1	0.771	0.653	0.154	∞ (75.2~∞)	*
	POHR	37°42′10.93″ N 128°36′05.77″ E	30	18	8.53	5	0.95	0.004	30	13.7	9.6	1	0.810	0.737	0.092	∞ (103.9~∞)	*
	YOHR	37°07′33.03″ N 128°50′03.57″ E	31	12	5.93	0	0.85	0.004	30	11.7	8.7	0	0.798	0.759	0.050	241.8 (77.1~∞)	*
	DNHR	37°02′19.48″ N 128°30′46.47″ E	25	8	5.41	2	0.88	0.002	29	10.9	8.3	0	0.778	0.685	0.122	∞ (120.3~∞)	*
	DDHR	36°53′44.40″ N 128°18′28.29″ E	30	9	5.12	1	0.83	0.003	28	12.4	9.2	1	0.805	0.687	0.149	762.4 (79.0~∞)	*
	BDHR	36°32′16.18″ N 127°50′32.45″ E	34	3	1.35	0	0.53	0.001	28	9.3	7.2	3	0.767	0.695	0.096	∞ (122.3~∞)	*
	WJHR	37°15′04.23″ N 128°04′58.20″ E	29	15	7.22	3	0.91	0.003	30	13.4	9.6	0	0.828	0.750	0.099	79.0 (24.4~137.5)	*
	JYHR	37°25′36.64″ N 128°33′48.47″ E	30	17	8.16	3	0.94	0.002	30	13.9	9.7	4	0.833	0.705	0.155	∞ (149.3~∞)	*
	PHHR	37°39′23.67″ N 128°19′56.68″ E	26	15	7.63	6	0.92	0.002	31	14.3	10.0	2	0.842	0.781	0.074	∞ (156.5~∞)	NS
	WSHR	37°30′03.74″ N 127°59′38.31″ E	4	4	—	2	—	—	4	4.7	—	0	—	—	—	∞ (17.1~∞)	—
	PSHR	37°40′15.34″ N 128°27′50.82″ E	12	6	5.00	4	0.68	0.001	12	9.4	9.4	2	0.848	0.787	0.075	∞ (∞~∞)	*
	CSHR	38°14′36.27″ N 127°27′45.55″ E	28	13	6.75	10	0.90	0.003	—	—	—	—	—	—	—	—	—
East‐flowing rivers	YOHE	38°04′36.86″ N 128°27′10.91″ E	28	5	2.85	1	0.57	0.002	30	10.1	7.9	1	0.797	0.730	0.086	55.4 (30.7~∞)	*
	GYHE	37°50′53.31″ N 128°43′27.48″ E	60	3	0.40	0	0.07	< 0.001	56	5.8	4.7	2	0.689	0.591	0.147	32.4 (18.1~42.9)	*
	YNHE	38°02′20.39″ N 128°35′48.81″ E	4	2	—	1	—	—	4	4.2	—	0	—	—	—	∞ (3.1~∞)	—
	UGHE	35°35′40.88″ N 129°14′00.16″ E	19	7	4.28	3	0.67	0.014	20	9.7	7.9	1	0.699	0.537	0.240	15.7 (8.0~61.8)	*
Geum River	BSGR	36°29′26.34″ N 127°51′54.82″ E	29	6	2.82	4	0.48	0.001	27	7.2	5.6	5	0.705	0.662	0.059	51.2 (22.2~196.1)	*
	MWGR	35°54′11.38″ N 127°47′40.56″ E	29	6	3.59	2	0.76	0.003	29	9.7	7.5	2	0.703	0.649	0.050	∞ (81.9~∞)	*
	ZkBOGR	36°24′40.64″ N 126°37′35.75″ E	32	4	2.26	3	0.63	0.003	30	6.3	5.0	0	0.507	0.559	0.048	138.1 (3.8~∞)	*
	ZtBOGR	36°24′40.64″ N 126°37′35.75″ E	23	7	4.50	6	0.77	0.007	30	5.0	3.9	0	0.395	0.278	0.300	1.0 (0.7~1.0)	*
Nakdong River	THNR	37°05′44.20″ N 129°01′43.65″ E	26	7	3.83	1	0.78	0.006	29	12.2	9.0	2	0.794	0.606	0.240	43.8 (15.7~1572.0)	*
	YSNR	36°57′49.11″ N 128°36′56.05″ E	26	5	2.89	2	0.71	0.003	30	8.7	6.6	2	0.681	0.611	0.104	52.5 (31.2~143.1)	*
	MGNR	36°48′41.99″ N 128°14′51.79″ E	34	4	1.67	1	0.43	0.002	30	6.9	5.4	0	0.644	0.605	0.061	13.1 (6.9~40.1)	*
	SYNR	36°32′14.08″ N 127°54′19.34″ E	19	7	4.15	2	0.70	0.004	30	8.3	6.3	2	0.682	0.444	0.352	3.9 (1.8~4.3)	*
	GSNR	35°49′42.41″ N 127°48′28.44″ E	22	2	0.55	0	0.09	0.002	30	5.0	4.3	0	0.623	0.553	0.098	14.0 (7.5~33.5)	*
	SSNR	35°17′42.01″ N 127°49′30.26″ E	33	9	5.37	6	0.86	0.011	30	9.0	6.6	5	0.684	0.487	0.291	7.4 (2.0~6.9)	*
	CGNR	36°13′34.99″ N 128°53′59.37″ E	2	1	—	1	—	—	—	—	—	—	—	—	—	—	—
Seomjin River	NWSR	35°23′16.90″ N 127°27′21.17″ E	29	6	3.26	5	0.74	0.005	30	7.0	5.6	15	0.630	0.565	0.097	∞ (89.8~∞)	*
Mangyeong River	WOMR	35°53′06.00″ N 127°14′35.42″ E	30	7	3.51	5	0.70	0.004	—	—	—	—	—	—	—	—	—
Total			754	122	—	—	0.96	0.014	717	30.4	—	—	0.882	0.627	0.290	∞ (75.2~∞)	*

Abbreviations: AR, allelic richness; BDHR, Boeun Dalcheon; BOGR, Boryeong Okgyecheon; BSGR, Boeun Samgacheon; CGNR, Cheongsong Gilancheon; CSHR, Cheorwon Sagokcheon; DDHR, Danyang Danyangcheon; DNHR, Danyang Namjicheon; FIS, inbreeding coefficient; GSNR, Geochang Sojeongcheon; GYHE, Gangneung Yeongokcheon; H, haplotype diversity; HE, expected heterozygosity; HO, observed heterozygosity; Hr, haplotype richness; HWE, *p* values for multilocus tests for Hardy–Weinberg Equilibrium (* < 0.05); IBHR, Inje Bukcheon; JYHR, Jeongseon Yongtancheon; MGNR, Mungyeong Geumcheon; MWGR, Muju Wondangcheon; N, sample sizes; NA, mean number of alleles across loci; NE, effective population size; NH, number of haplotypes; NWSR, Namwon Woncheoncheon; PA, number of private alleles; PH, number of private haplotypes; PHHR, Pyeongchang Heungjeong Valley; POHR, Pyeongchang Odaecheon; PSHR, Pyeongchang Soksacheon; SSNR, Sancheong Samjangcheon; SYNR, Sangju Yongyucheon; THNR, Taebaek Hwangjicheon; UGHE, Ulsan Guksucheon; WJHR, Wonju Jupocheon; WOMR, Wanju Odocheon; WSHR, Wonju Seomgang; YNHE, Yangyang Namdaecheon; YOHE, Yangyang Osaekcheon; YOHR, Yeongwol Okdongcheon; YSNR, Yeongju Sacheon; π, nucleotide diversity.

Genomic DNA was extracted from caudal fin tissue (~3 mm) using a P&C Animal Genomic DNA Kit (Biosolution, Republic of Korea), according to its protocol guidelines. The concentration of the extracted genomic DNA was determined using a NanoDrop UV/VIS spectrophotometer (Thermo Fisher Scientific, USA) and stored at −20°C until molecular experiments.

### 2.2 Mitochondrial DNA Sequencing

#### 2.2.1 Mitochondrial DNA Marker Sequencing

We used mtDNA COI and CR as molecular markers for phylogenetic and population genetic analyses. mtDNA COI (625 bp) was amplified by polymerase chain reaction (PCR) using FishF1 and FishR1, the universal primers developed for DNA barcoding for fishes (Ward et al. 2005). For mtDNA CR (832 bp), we newly developed *Z. koreanus* specific primers for this study (FW: 5′‐TCCCAAAGCCAGAATTCTAAATTAAAC‐3′; RV: 5′‐CTTCAGTGTTATGCTTTGTGTTAAG‐3′). PCR amplification was performed in a total reaction volume of 15 μL containing 10× Dream Taq Green buffer (Thermo Fisher Scientific, USA), 2.5 mM of each dNTP (Bio Basic Inc., Canada), 10 pmol of each of the forward and reverse primers, 0.2 units of *Taq* DNA polymerase (Thermo Fisher Scientific), and 3~43 ng/μL of genomic DNA template.

The following thermal cycling conditions for the COI (and CR) were applied: initial denaturation at 95°C for 2~3 min, ‘followed by 35 cycles of denaturation at 94°C for 30 s, annealing at 57°C for 30 s (CR; 60°C for 40 s) and extension at 72°C for 1 min’, and final extension at 72°C for 10 min. The amplified PCR products were checked on 2% agarose gels stained with Redsafe (iNtRON Biotechnology, Korea), then purified enzymatically by incubation using the Exonuclease I and Shrimp Alkaline Phosphatase (New England BioLabs, USA) at 37°C for 15 min, followed by inactivation by incubation at 85°C for 15 min. The purified mtDNA fragments were subjected to direct sequencing only in the forward direction using the same forward primers in an ABI3730xl automated DNA sequencer (Applied Biosystems, USA) at Macrogen Inc. (Seoul, Republic of Korea). The obtained COI and CR sequences of *Z. koreanus* and *Z. temminckii* were edited and aligned using Geneious Prime v.2022.2.1 (Biomatters Ltd., Auckland, New Zealand), and finally verified manually. By concatenating the aligned nucleotide sequences of both markers, a total of 1,457 bp was used for subsequent analyses.

#### 2.2.2 Mitochondrial Whole Genome (Mitogenome) Sequencing

To compare and analyze mitochondrial DNA genomes of *Z. koreanus* populations from each river basin, genomic DNA was isolated for three *Z. koreanus* individuals (one individual each from the Han, Geum, and Nakdong Rivers) using the P&C Animal Genomic DNA kit (Biosolution) according to the protocol guidelines. We quantified the level of genetic divergence at the whole mitogenome level between different riverine populations of *Z. koreanus* together with *Z. temminckii* . Paired‐end sequencing of the mitogenomes of the extracted DNA was performed on the Miseq platform (Illumina Inc., San Diego, CA). We obtained 3,748,852 (Han River), 3,417,854 (Geum River), and 3,614,134 (Nakdong River) raw read pairs with a length of 301 bp, and were trimmed using Geneious Prime 2022.1.1 (Biomatters Ltd.) by error probability limit set to 5%. The assembly and annotation of the mitogenome were also accomplished using the Geneious Prime software. We then compared their sequences with a previously published mitochondrial genome database of *Z. koreanus* that was originated from the Seomjin River [accession no. NC025286 (Chen et al. 2016)]. The assembled *Z. koreanus* mitogenomes were further compared with that of the closely related species *Z. temminckii* [AP012116 (Miya et al. 2015)]. Also, mitochondrial genome map was completed using OrganellarGenomeDRAW (OGDRAW) ver. 1.3.1 (Greiner et al. 2019).

### 2.3 Microsatellite Marker Development and Genotyping

To develop novel microsatellite markers for *Z. koreanus*, whole nuclear genome sequence information was utilized. For this, a total of 1 μg of gDNA from an individual of *Z. koreanus* collected from the IBHR population (Inje Bukcheon; Table 1) was sheared using the S220 Ultra sonicator (Covaris, Woburn, MA.). Library preparation was performed with the MGIEasy DNA library prep kit (MGI, China) according to the manufacturer's instructions. Briefly, after size‐selection of fragmented gDNA using AMPure XP magnetic beads, the fragmented gDNA was end‐repaired and A‐tailed using the enzyme mix included in the MGIEasy Universal DNA Library Prep Set (MGI, China) according to the manufacturer's protocol at 37°C for 30 min, and 65°C for 15 min. Indexing adapter was ligated to the ends of the DNA fragments at 23°C for 60 min. After cleanup of adapter‐ligated DNA, PCR was performed to enrich those DNA fragments that had adapter molecules. Thermocycler conditions were as follows 95°C for 3 min, ‘7 cycles of 98°C for 20 s, 60°C for 15 s, and 72°C for 30 s’, with a final extension at 72°C for 10 min. The double stranded library was quantified using the QuantiFluor ONE dsDNA System (Promega, USA). The library was circularized at 37°C for 30 min, and then digested at 37°C for 30 min, followed by cleanup of circularization product. To make DNA nanoball (DNB), the library was incubated at 30°C for 25 min using DNB enzyme included in the DNBSEQ‐G400RS High‐throughput Sequencing Set (FCL PE150) V3.1 (MGI, China). Finally, the library was quantified by the QuantiFluor ssDNA System (Promega). Sequencing of the prepared DNB was conducted on the MGIseq system (MGI) with 150 bp paired‐end reads.

A total of 25 primer pairs for species‐specific microsatellite loci of *Z. koreanus* were obtained using PRIMER3 (Untergasser et al. 2012). Each locus was tested by PCR amplification in a total volume of 15 μL containing 2.5 mM of each dNTP, 10× Dream Taq Green buffer, 10 pmol of each primer, 0.2 units of *Taq* DNA polymerase and 3~43 nL/μL of genomic DNA. PCR conditions were as follows: initial denaturation at 95°C for 3 min; followed by 35 cycles of denaturation at 94°C for 1 min, annealing at 53°C–56°C for 30 s, extension at 72°C for 1 min, and a final extension at 72°C for 20 min. Twenty‐five loci were tested through PCR amplification, of which nine loci were finally selected for population genetic screening based on polymorphism and reproducibility. Forward primers were labeled with fluorescent dyes such as FAM, HEX, or TAMRA. PCR amplification was performed as described for mtDNA and reaction conditions for each primer pair were as described above. PCR products were verified after the PCR reaction and electrophoresed on an ABI 3730xl automated DNA sequencer (Applied Biosystems). Amplified fragment sizes were determined with the ROX 500 bp size standard (ABI) using GENEMAPPER software v5.0 (Applied Biosystems).

### 2.4 Genetic Diversity

To investigate differences in the level of mitochondrial genetic diversity among 29 populations, including 28 *Z. koreanus* and one *Z. temminckii* populations, the number of haplotypes (*N*_H_), haplotype diversity (*H*), nucleotide diversity (*π*), and number of private haplotypes (PH) were calculated using ARLEQUIN v3.5 (Excoffier and Lischer 2010). Also, haplotype richness (*Hr*) corrected for unbalanced sample sizes among the samples was calculated using CONTRIB v1.02 (Petit et al. 1998). The Kruskal–Wallis test was performed to examine whether there was a significant difference in the level of *Hr* between the Han River (*N* = 11), East‐flowing rivers (*N* = 3), Geum River (*N* = 3), and Nakdong River (*N* = 6) for *Z. koreanus* populations. The three populations (WSHR, YNHE, and CGNR) of *Z. koreanus* were omitted from this and subsequent analyses (except for the phylogenetic analyses) because of insufficient sample sizes (*N* < 5).

To assess levels of microsatellite diversity in *Z. koreanus* and *Z. temminckii* from South Korea, the mean number of alleles per locus (*N*_A_), allelic richness (AR) corrected for unequal sample sizes, observed (*H*_O_) and expected (*H*_E_) heterozygosities, inbreeding coefficient (*F*_IS_), and deviation from Hardy–Weinberg equilibrium (HWE) were calculated using GENEPOP v4.0 (Rousset 2008) and FSTAT v2.9.3.2 (Goudet 2001). The 95% significance levels for the exact test for HWE were adjusted using a Bonferroni correction. Contemporary effective population sizes (*N*_E_) were estimated for each population using NeESTIMATOR ver. 2.10 software (Do et al. 2014) based on the linkage disequilibrium (LD) method.

### 2.5 Phylogeographic Relationships

To determine phylogeographic relationships among *Z. koreanus* populations in geographically separated river basins, phylogenetic trees were reconstructed with maximum likelihood (ML) methods as implemented in Mega X (Kumar et al. 2018). We used the concatenated DNA fragment (1,457 bp) of COI and CR to make the sequence length the same as that of the outgroup for performing the phylogeographic analysis. Sequences of *Zacco platypus* as outgroup were retrieved from GenBank (accession no.: NC023105), and statistical support was estimated by 1000 bootstrap replicates. To determine the mtDNA sequence divergence between the six river basins within *Z. koreanus* and also between *Z. koreanus* and *Z. temminckii* , pairwise genetic distances were estimated using a Kimura‐2‐Parameter (K2P) distance model, with 1000 bootstrap replications, using MEGA X. A haplotype network was constructed with HAPSTAR v0.7 (Teacher and Griffiths 2011) to infer phylogenetic relationships among haplotypes.

### 2.6 Population Genetic Structure

To assess the degree of genetic differentiation within *Z. koreanus* and also between *Z. koreanus* and *Z. temminckii* based on mtDNA and microsatellites, calculations of pair‐wise *F*_ST_ estimates among 26 populations were performed with ARLEQUIN and GENEPOP. The 95% significance levels for the pairwise population comparisons were adjusted using a Bonferroni correction. The population genetic structure analysis was further performed using a Bayesian model‐based clustering algorithm implemented in STRUCTURE v2.3.4 under a model of admixed ancestry among populations and correlated allele frequencies (Pritchard et al. 2000). STRUCTURE calculates a likelihood score when the data are forced into a set number of genetic clusters (*K*) = 1–26. We applied 10 iterations, with 100,000 burn‐in steps followed by 1,000,000 Markov Chain Monte Carlo (MCMC) generations. The best‐supported number of genetic clusters (*K* value) was determined by using the delta *K* (Δ*K*) method implemented in the web‐based tool STRUCTURE HARVESTER (http://taylor0.biology.ucla.edu/structureHarvester/), based on the rate of change in the log probability of data between successive *K* values (Earl and Vonholdt 2012). To further examine whether hierarchical genetic structure exists among river basins and between species, we additionally examined STRUCTURE results up to *K* = 6, as well as *K* = 21, which showed the second highest Δ*K* value (Figure S1). In addition, we estimated patterns of genetic differentiation between populations using principal coordinates analysis (PCoA) using GenAlEx v6.51 (Peakall and Smouse 2006).

## 3 Results

### 3.1 Genetic Diversity

A total of 122 mtDNA (COI and CR) haplotypes (1,457 bp) were identified among 754 individuals from 28 populations of *Z. koreanus* and one population of *Z. temminckii* (Table 1). Genetic diversity indices, including *N*_H_, *Hr*, *H*, *π* and PH within the 29 populations were estimated and are summarized in Table 1. The range of *H* with a *Z. koreanus* population was 0.07 (GYHE) to 0.95 (POHR) and that of *π* from 0.000 (GYHE) to 0.014 (UGHE). *Hr* values for each river basin within *Z. koreanus* ranged from 1.35 (BDHR) to 8.53 (POHR) in the Han River, 0.40 (GYHE) to 4.28 (UGHE) in East‐flowing rivers, 2.26 (ZkBOGR) to 3.59 (MWGR) in the Geum River, 0.55 (GSNR) to 5.37 (SSNR) in the Nakdong River, 3.26 (NWSR) in the Seomjin River, and 3.51 (WOMR) in the Mangyeong River, respectively. The Han River populations thus had a significantly higher level of *Hr* than the other riverine populations (Kruskal–Wallis test, *p* = 0.014). *Z. temminckii* had seven haplotypes, including six private haplotypes, among 23 individuals, and *Hr*, *H* and *π* values were found to be 4.50, 0.77 and 0.007, respectively. GenBank accession numbers for the determined 122 mtDNA haplotypes are PV340652–PV340773.

The mean number of microsatellite alleles (*N*_A_) per *Z. koreanus* population was 9.4, ranging from 4.2 (YNHE) to 14.3 (PHHR). The AR values within *Z. koreanus* ranged from 4.3 (GSNR) to 10.0 (PHHR; mean = 0.739), and *H*_E_ and *H*_O_ ranged from 0.587 (ZkBOGR) to 0.848 (PSHR) and from 0.444 (SYNR) to 0.787 (PSHR; mean = 0.645), respectively. The *N*_A_ and AR values of *Z. temminckii* were observed to be 5.0 and 3.9, indicating a lower level of diversity than those of *Z. koreanus* populations. Additionally, PA of *Z. koreanus* ranged from 0 to 15, and that of *Z. temminckii* was 0. The range of *F*_IS_ values was from 0.048 (ZkBOGR) to 0.352 (SYNR), and *H*_O_ of all populations (including *Z. temminckii* ) was less than *H*_E_, suggesting the possibility of nonrandom mating (i.e., inbreeding). Excluding *Z. temminckii* , *N*_E_ values of *Z. koreanus* populations were often unbounded or high, with a 95% confidence interval (CI) ranging from 75.2 to ∞ (Table 1).

### 3.2 Phylogeographic Relationships of *Z. koreanus* Among Six River Basins With *Z. temminckii*

**FIGURE 2:**
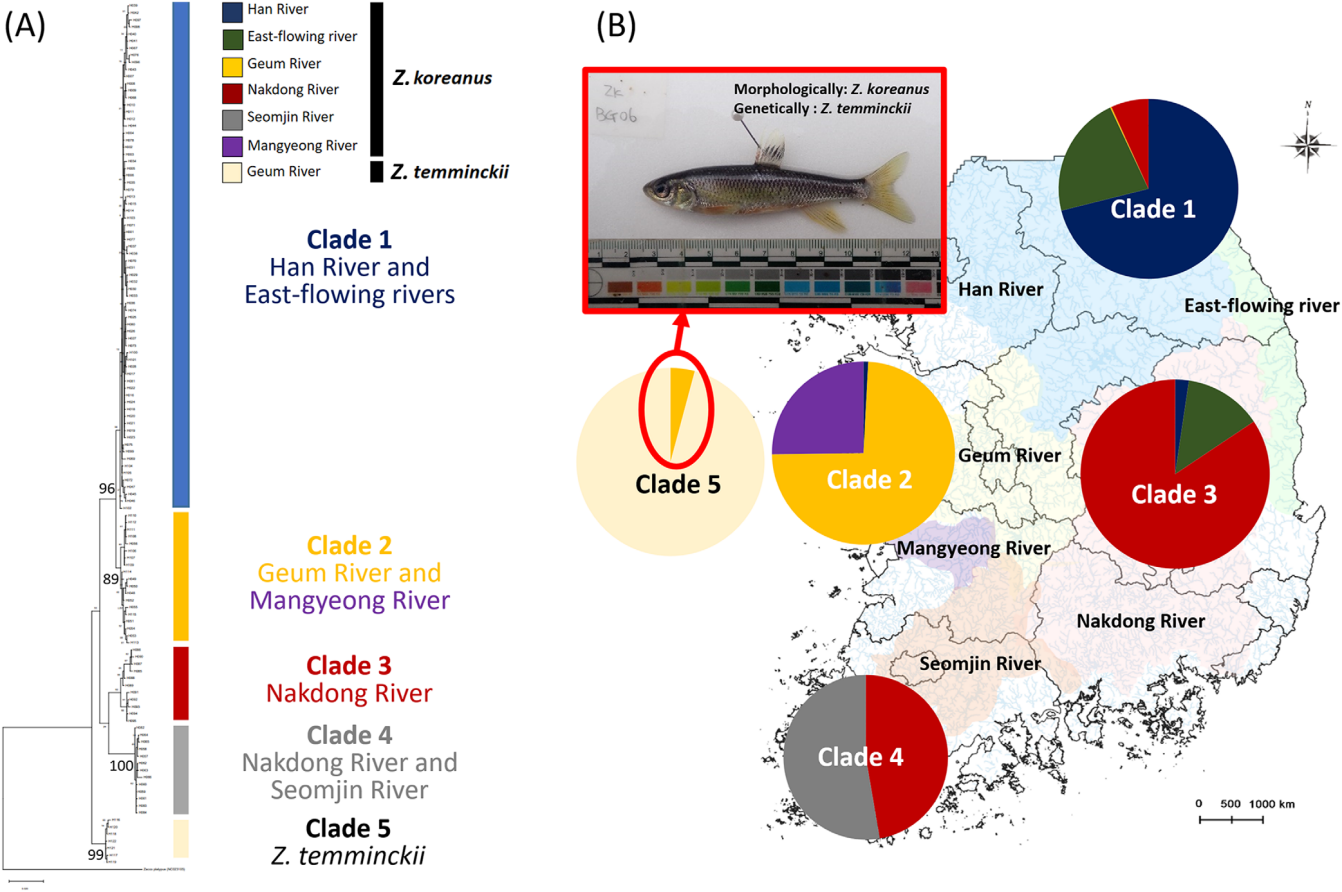
Phylogenetic relationships among 122 haplotypes (H01‐122) based on concatenated mitochondrial DNA COI and CR sequences (1,457 bp). (A) Five well‐separated clades included; Clade 1, mainly composed of the Han River and the East‐flowing rivers, Clade 2, the Geum River, Clade 3, the Nakdong River, Clade 4 the Seomjin River, and Clade 5 *Zacco temminckii* . The numbers at the nodes represent the bootstrap support values. (B) Distribution of each lineage by river basins. One individual with morphologically *Zacco koreanus* but genetically *Z. temminckii* at mtDNA is shown.

**FIGURE 3:**
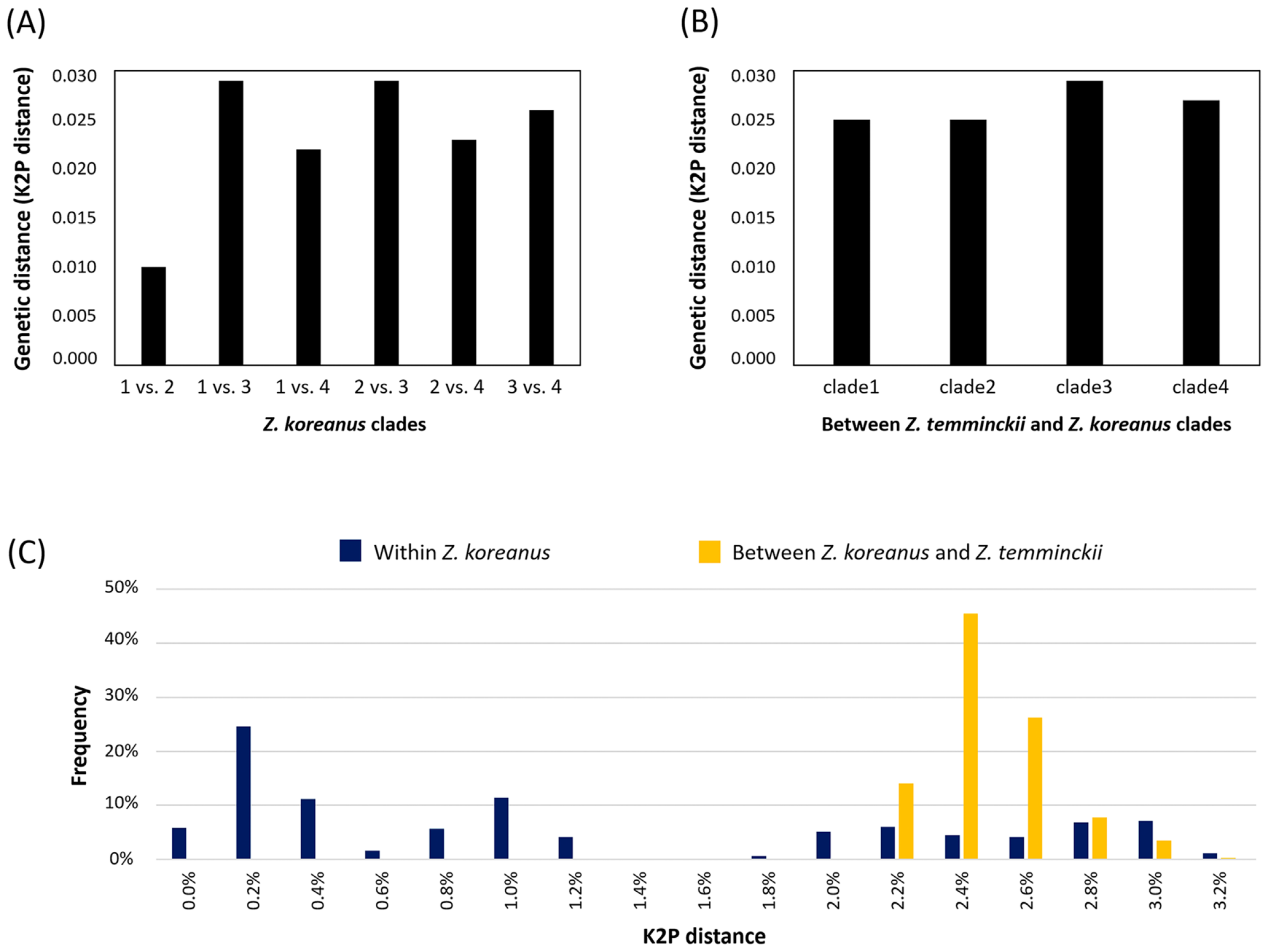
Genetic distances (K2P) between five clades identified from mitochondrial DNA COI and CR sequences (1,457 bp). (A) Genetic distances between four clades of *Zacco koreanus*, as shown in Figure 2. (B) Genetic distances between four lineages of *Z. koreanus* and *Z. temminckii* . (C) Barcoding gap based on intra‐ and interspecific K2P genetic distances.

Phylogenetic analysis using 122 mtDNA haplotypes identified a total of five major mtDNA clades, including four *Z. koreanus* lineages and one *Z. temminckii* lineage (Figure 2A). The distribution of these clades corresponded generally to major river basins. Clade 1 was primarily associated with the Han River and East‐flowing rivers, Clade 2 with the Geum and Mangyeong rivers, Clade 3 with the Nakdong River, and Clade 4 with the Nakdong and Seomjin rivers. *Z. temminckii* formed a separate lineage (Clade 5). Nevertheless, one haplotype shared between *Z. koreanus* and *Z. temminckii* was identified. More specifically, one *Z. koreanus* individual with a *Z. temminckii* mtDNA lineage was identified—a morphologically *Z. koreanus* individual had a *Z. temminckii* mtDNA haplotype (Clade 5; Figure 2B). The mtDNA genetic distances (K2P distances) between the four clades within *Z. koreanus* were—Clade 1 vs. Clade 2 = 1.0%, 1 vs. 3 = 2.9%, 1 vs. 4 = 2.2%, 2 vs. 3 = 2.9%, 2 vs. 4 = 2.3%, and 3 vs. 4 = 2.6%, respectively (Table 2, Figure 3A). The Nakdong and Seomjin rivers populations represented a distinct lineage with a mean of 2.6% (2.2%~2.9%) genetic distance from other lineages, which is similar to the level of genetic distance with *Z. temminckii* (2.7%; 2.5%~2.9%; Figure 3B). Overall, the intraspecific mtDNA genetic distance within *Z. koreanus* (0.0%~3.2%) overlapped with the interspecific distance with *Z. temminckii* (2.2%~3.0%), indicating some degree of ambiguity in molecular‐based species delimitation (Figure 3C).

**TABLE 2:** Pairwise Kimura 2‐parameter (K2P) genetic distances among the five clades of *Zacco koreanus* and *Z. temminckii* based on mitochondrial DNA COI and CR sequences. Clade 1: *Z. koreanus* from the Han and East‐flowing rivers; Clade 2: *Z. koreanus* from the Geum and Mangyeong rivers; Clade 3: *Z. koreanus* from the Nakdong River; Clade 4: *Z. koreanus* from the Nakdong and Seomjin rivers; Clade 5: *Z. temminckii* .

	Clade 1	Clade 2	Clade 3	Clade 4
Clade 2	0.010			
Clade 3	0.029	0.029		
Clade 4	0.022	0.023	0.026	
Clade 5	0.025	0.025	0.029	0.027

**FIGURE 4:**
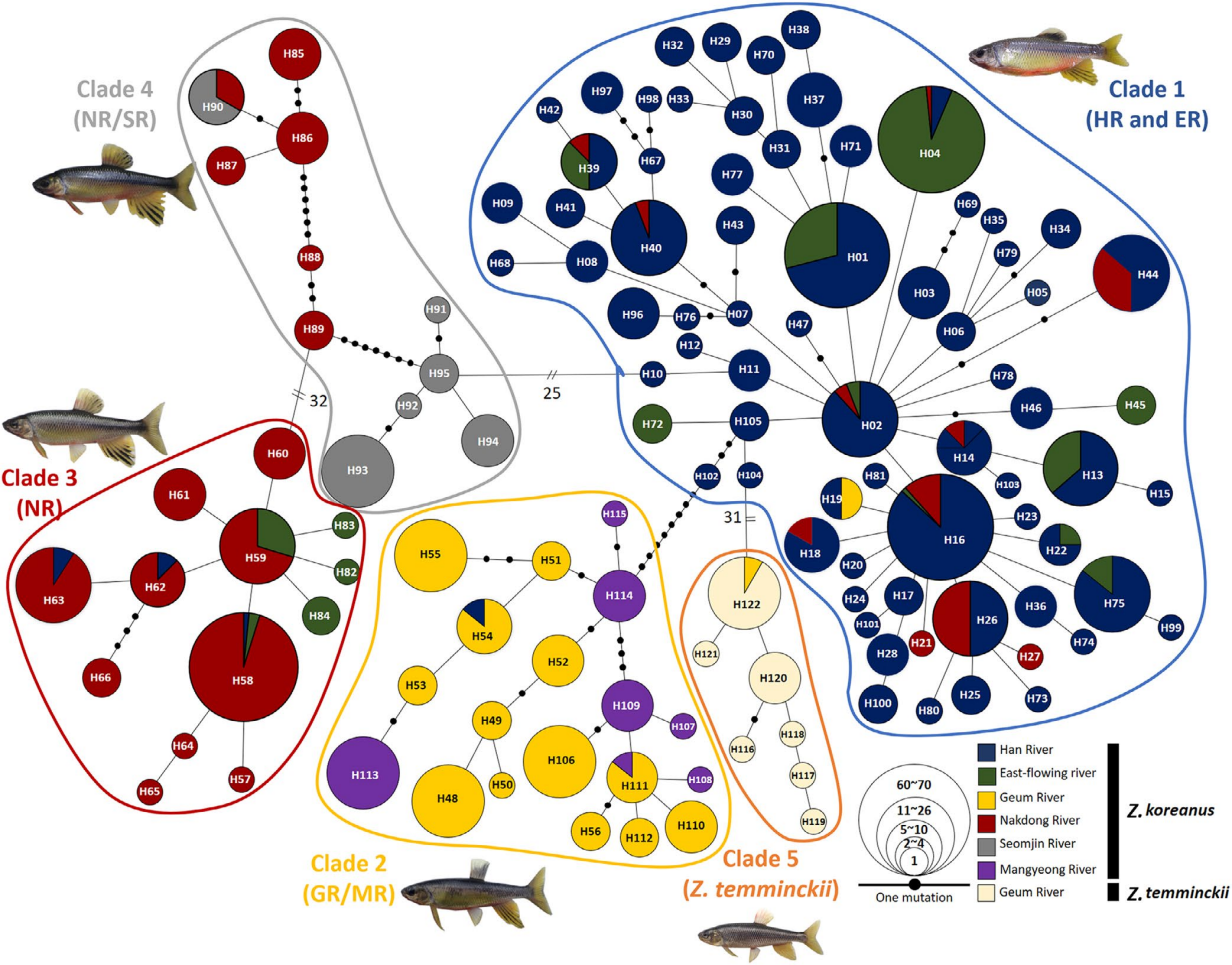
Haplotype network of 122 haplotypes (H01‐122) identified based on concatenated mitochondrial COI and CR sequences (1,457 bp). Each line in the network represents a single mutational step between the haplotypes, irrespective of its length. Different colors denote the respective geographic regions. The area of the circles is proportional to individual numbers found for the respective haplotypes. The small, filled circles denote intermediate haplotypes that were not present in our samples, but were necessary to connect all of the observed haplotypes within the network.

Similar to the findings of phylogenetic analysis, the haplotype network also identified five distinct haplotype groups, with all haplotypes connected by 1~110 mutational steps (Figure 4). The HR and HE populations shared a majority of haplotypes (Clade 1). The genetic divergence among the five haplotype groups ranged from nine to 100 mutational steps, with the smallest difference observed between Clade 1 and Clade 2 and the largest difference between Clade 3 and Clade 5. Despite the large genetic divergences between the five distinct groups, some populations shared haplotypes with different lineages (Figure 4) (e.g., H02, H04, H14, H16, H19).

**FIGURE 5:**
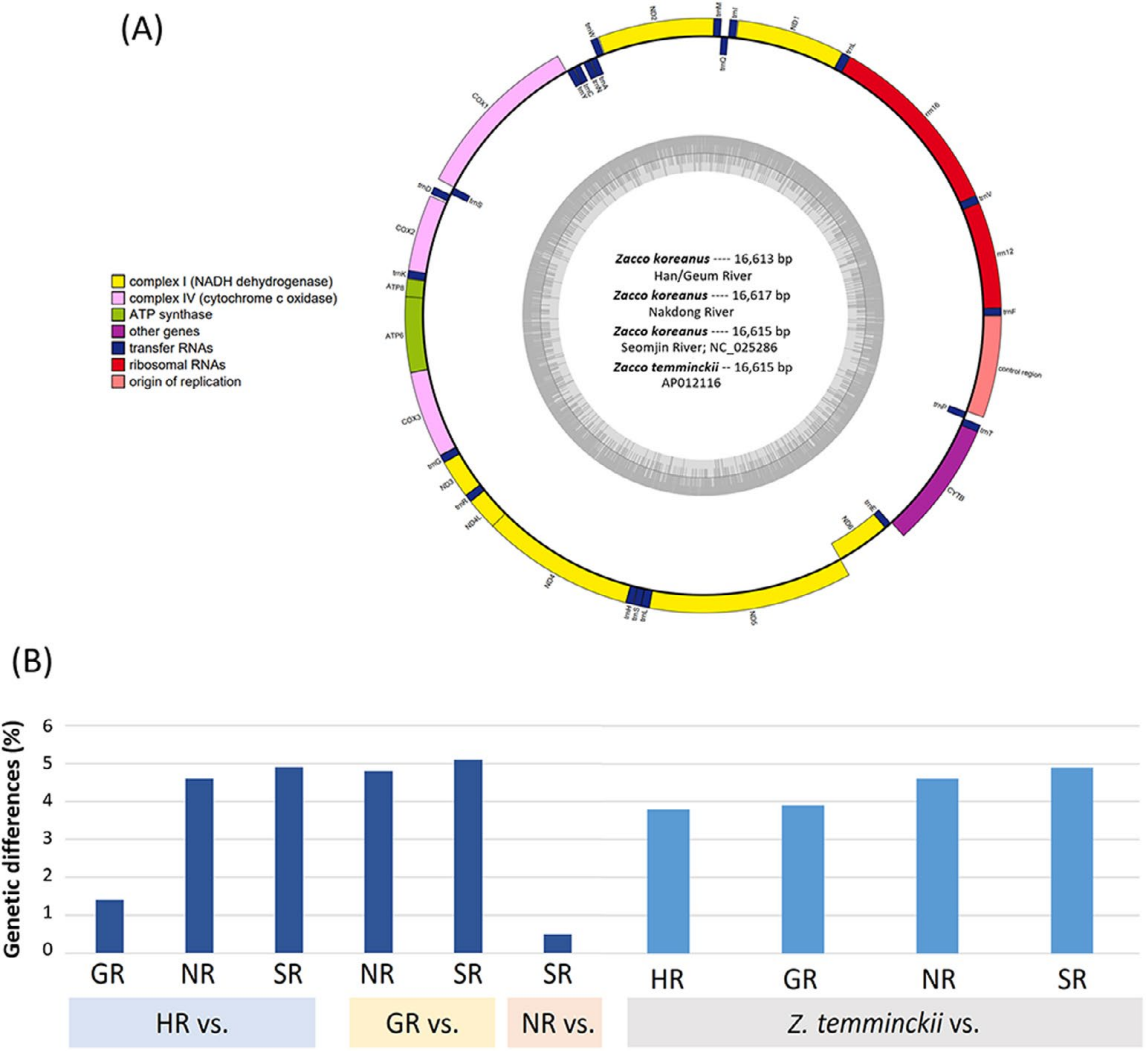
(A) Map of the mitogenome of *Zacco koreanus* in each river basin. Genes belonging to different functional groups are color coded. The dark‐gray inner circle represents GC content, and the light‐gray inner circle represents AT content. (B) Comparisons of genetic differences between river basins of *Z. koreanus* and also between *Z. koreanus* and *Zacco temminckii* based on whole mitogenome sequences.

The mitogenomes of *Z. koreanus* from the Han and Geum Rivers were assembled to 16,613 bp, and those from the Nakdong River to 16,617 bp (Figure 5A). The assembled HR/GR/NR mitogenomes of *Z. koreanus* were composed of overall 45.4%/43.0%/43.1% GC content and A—30.6%/30.5%/30.0%, C—26.4%/26.4%/26.1%, G—16.6%/16.6%/17.1% and T—26.4%/26.4%/26.8% nucleotides, respectively. All three mitogenomes consisted of 13 protein‐coding genes (PCGs), 22 tRNA genes, and two ribosomal RNA (rRNA) genes, which were observed to be the same in number and order to the published mitochondrial genome databases of *Z. koreanus* (GenBank accession no. NC_025286) and *Z. temminckii* (AP012116), suggesting that no structural variations such as insertions or rearrangements had occurred. When comparing the five mitogenome sequences of *Z. koreanus* and *Z. temminckii* , there was a mean genetic distance of 3.6% (0.5%~5.1%) within *Z. koreanus* and a mean of a 4.3% (3.8%~4.9%) with *Z. temminckii* , suggesting again that degrees of intra‐ and interspecific genomic divergences overlapped (Figure 5B).

### 3.3 Genetic Differentiation and Population Structure

**FIGURE 6:**
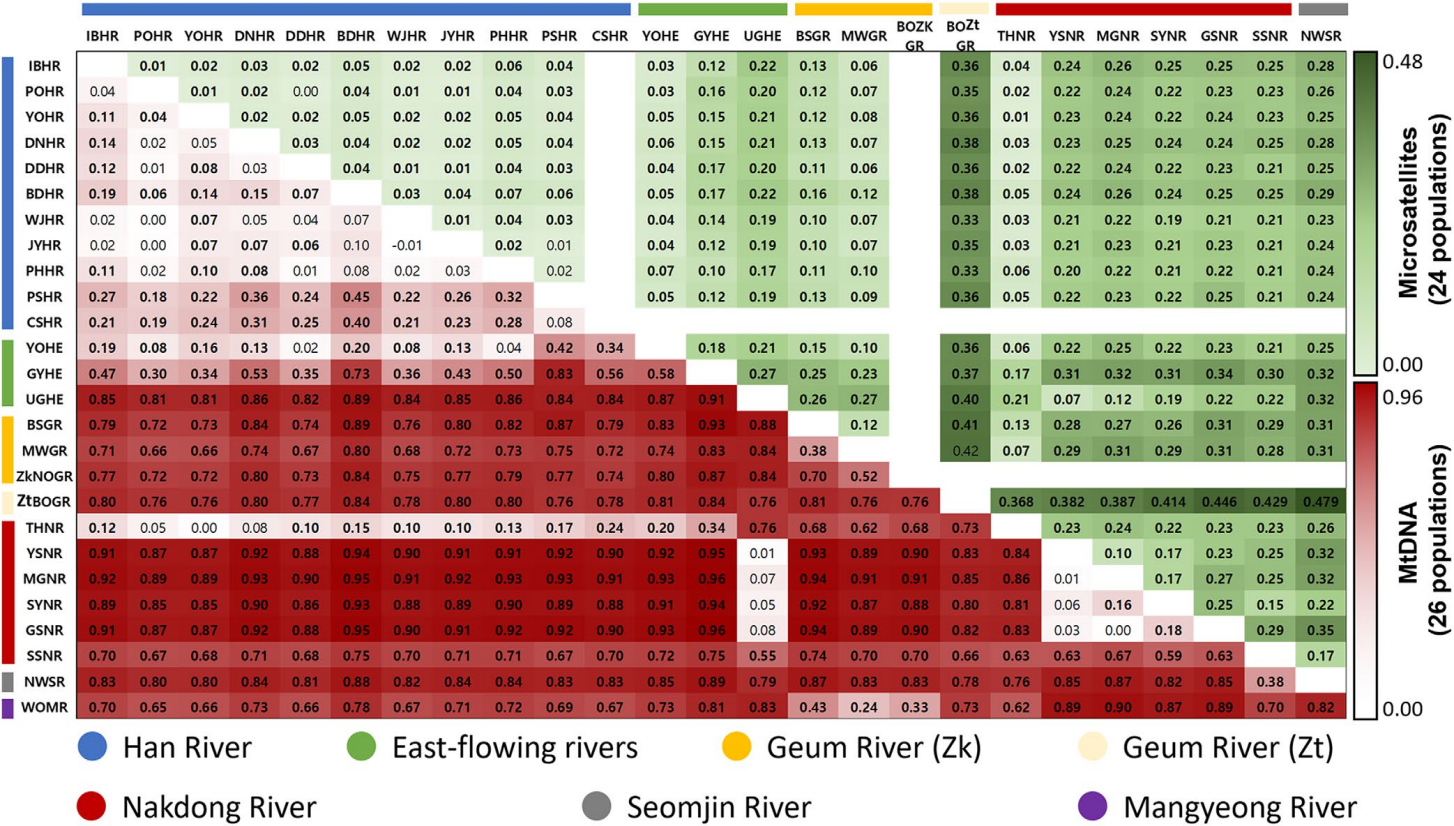
Pairwise genetic differentiation (*F*_ST_) between 25 populations of *Zacco koreanus* and one *Zacco temminckii* (ZtBOGR), based on concatenated mitochondrial DNA COI and CR sequences (1,457 bp) above diagonal and nine microsatellite loci below diagonal. Significant pairwise and *p* values are shown in bold (*p* < 0.05) after a Bonferroni correction.

The pairwise *F*_ST_ estimates for 26 populations of *Z. koreanus* ranged from −0.01 to 0.96 for mtDNA, and 0.00 to 0.35 for microsatellites, indicating generally high levels of genetic differentiation (except 36 pairwise comparisons) (Figure 6). The *F*_ST_ values of *Z. koreanus* with *Z. temminckii* were observed to be 0.66~0.85 (mtDNA) and 0.33~0.48 (microsatellites), suggesting moderately higher levels than those within *Z. koreanus* (Figure 6). Significant *F*_ST_ values were found not only between different river basins but also within the same river basins, although genetic differentiation levels within the same rivers were generally lower. Higher genetic differentiation was observed between river basins (Figure 6), indicating genetic differentiation and suggesting limited gene flow taking place among river basins due to geographic isolation. The levels of genetic differentiation were generally higher for the Nakdong and Seomjin Rivers than for the other river basins, suggesting that those riverine populations were more isolated from the other populations due to geographical or physical barriers.

**FIGURE 7:**
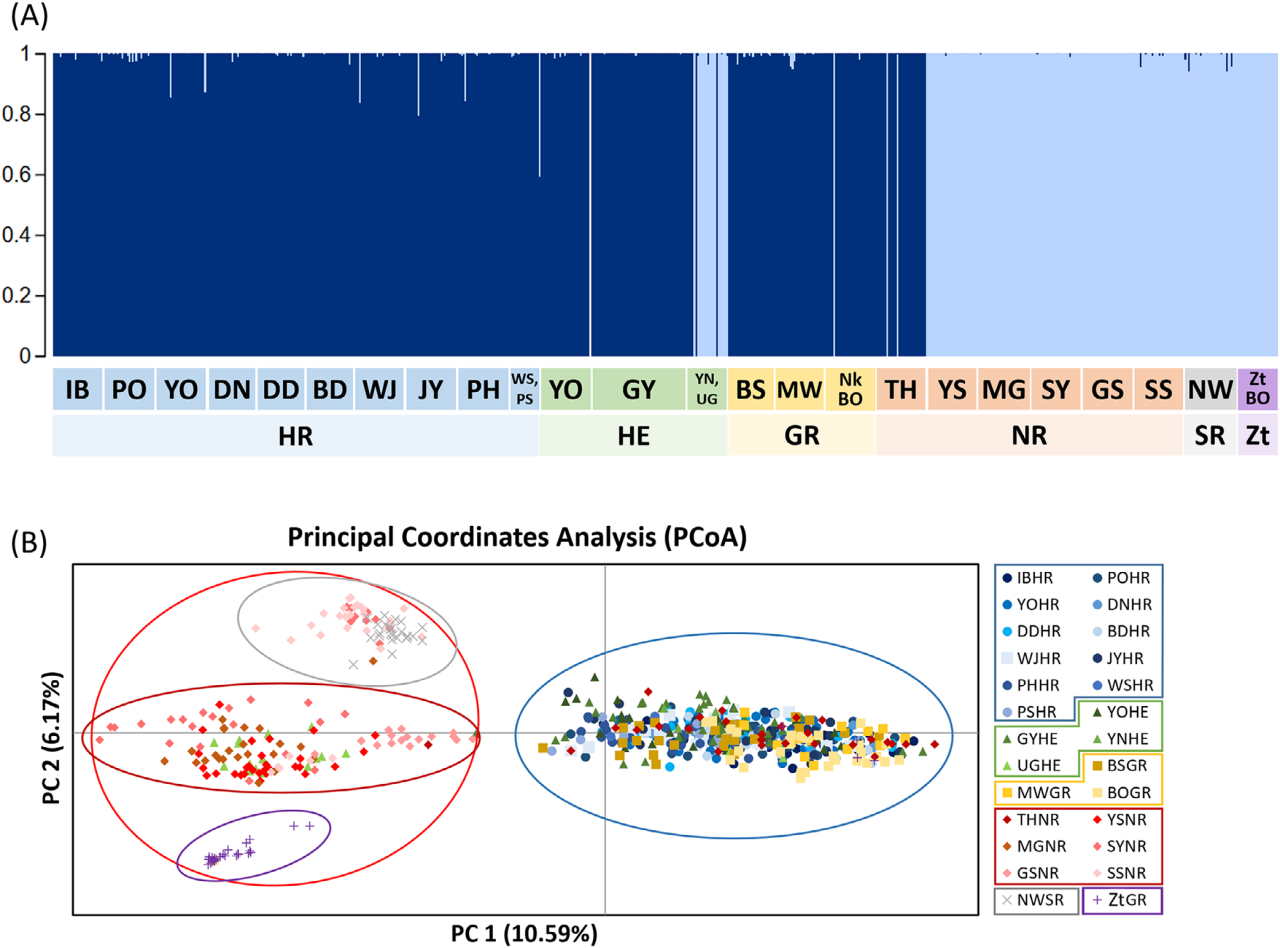
Population genetic structure of *Zacco koreanus* and *Z. temminckii* based on frequencies of genotypes at nine microsatellite loci. (A) Each individual is represented as a vertical bar partitioned into segments of different colors according to the proportion of the genome belonging to each of the identified clusters by STRUCTURE software. Analyses of population structure for all 26 populations at *K* = 2. (B) Results of principal coordinate analysis (PCoA) based on nine microsatellite genotypes for the 25 populations of *Z. koreanus* and one population (ZtBOGR) of *Z. temminckii* .

The results of STRUCTURE analysis based on the microsatellite data of the 26 populations, including the 25 *Z. koreanus* and one *Z. temminckii* , showed greatest support for two genetic clusters (*K* = 2) (Figure 7A; Figure S1). The population genetic structure results suggest that the Han, East‐flowing, and Geum river populations composed one cluster, while the Nakdong and Seomjin river populations composed another genetic cluster. The UGHE and THNR populations had genetic contributions from different river basins. Interestingly, *Z. temminckii* was with the same genetic cluster as the Nakdong and Seomjin River populations of *Z. koreanus*, which suggests the possibility of genetic exchange between the two species, as Nakdong and Seomjin Rivers are ecosystems where both species co‐occur (Kim, Han, et al. 2020). At *K* = 3–6, individuals were largely assigned to clusters corresponding to major geographic groups, although some individuals showed mixed ancestries (Figure S2). In contrast, at *K* = 21, individuals showed highly mixed assignment proportions across clusters, and no clear geographic grouping corresponding to river basins was observed (Figure S2).

In the PCoA analysis, two groups were identified (Han River/East‐flowing rivers/Geum River and Nakdong River/Seomjin River/Zt), where *Z. temminckii* was clustered with Nakdong and Seomjin Rivers populations of *Z. koreanus*. However, some populations from the Nakdong and Seomjin Rivers of *Z. koreanus* tended to form another group, while *Z. temminckii* formed a separate cluster (Figure 7B).

## 4 Discussion

### 4.1 Phylogenetic Relationships Among/Within *Z. koreanus* Populations and Between *Z. temminckii*

Previous studies of molecular systematics on *Zacco* species (later revised as genus *Nipponocypris*) in Korea suggested that *Z. platypus* first evolved from the most recent common ancestor in this particular lineage, and that *Z. koreanus* later diverged from *Z. temminckii* (Oh and Park 2009). The results of our phylogeographic analyses show that *Z. koreanus* populations in the Korean Peninsula are composed of four distinct matrilineal lineages (Clade 1: Han River, East‐flowing rivers; Clade 2: Geum River; Clade 3: Nakdong River; Clade 4: Nakdong and Seomjin River), while *Z. temminckii* has a different and ancestral lineage (Clade 5; Figure 2). Interestingly, however, we find one individual of morphologically *Z. koreanus* [e.g., no distinct red spot above the eye, yellowish sides, and red front edge of the pectoral fin (Kim et al. 2005; Chae and Yoon 2006)], but genetically *Z. temminckii* at mtDNA, suggesting the possibility of a natural hybrid between the two species or phenotypic plasticity leading to the similar morphology evolved within the species occurring in the same environments. Actually, the possible existence of natural hybrids between *Z. koreanus* and *Z. temminckii* has been suggested by previous molecular‐based phylogenetic investigations (Kim, Han, et al. 2020). Natural hybridization has been reported more frequently in freshwater fishes than in any other vertebrate groups, which may be associated with external fertilization in the aquatic environment (Scribner et al. 2000; Schwenk et al. 2008; Zbinden et al. 2023). Nevertheless, given the very small number of presumed hybrid individuals detected, further analysis is needed using more samples and also more molecular markers (e.g., nuclear genes, genome‐wide SNPs [single nucleotide polymorphisms]).

The mtDNA‐based phylogenetic and haplotype network analyses suggest that distinct lineages have evolved in the Nakdong and Seomjin River populations of *Z. koreanus*, leading to genetic distances of 2.2%~2.9% (mean = 2.6%) relative to the other lineages. These genetic distance levels are indeed similar to those (mean = 2.7%; 2.5%~2.9%) between *Z. koreanus* and *Z. temminckii* populations. In general, > 2%~3% molecular divergences have been suggested as suitable for delimitating species boundaries in animals (Hebert et al. 2003). The results provide evidence supporting the interpretation that the species boundary between *Z. koreanus* and *Z. temminckii* is unclear, as the two species may hybridize more freely than previously assumed. However, in a molecular phylogenetic study of *Z. temminckii* , *Z. koreanus*, and *Z. platypus* using the mtDNA cytochrome *b* (cyt *b*) gene, the genetic distance between *Z. temminckii* and *Z. koreanus* was estimated to be approximately 4.5% on the neighbor‐joining phylogenetic tree (Oh and Park 2009). The observed levels of divergences of the Nakdong and Seomjin River clades within *Z. koreanus* suggest that South Korean *Z. koreanus* consist of at least two cryptic species complex or an early stage of divergence within the species (Hebert et al. 2003; Wang et al. 2019; Kang et al. 2020; Kim, Jang, et al. 2020). The results of comparing the mitogenomes from different rivers also showed that the Nakdong and Seomjin River populations had a genetic distance of 4.7%–5.1% (Figure 5B). This level of divergence suggests that South Korean *Z*. *koreanus* may comprise deeply differentiated evolutionary lineages and raises the possibility that cryptic species diversity exists within what is currently recognized as a single species. The cryptic species complex represents a species that has little difference in morphology, but is likely to differentiate into potentially distinct species due to considerable genetic differences (Struck et al. 2018).

Nevertheless, the mitochondrial and nuclear datasets revealed partially incongruent patterns of genetic differentiation in *Z. koreanus*—the presence of five mitochondrial lineages, but only two major microsatellite clusters. Such mito–nuclear discordance has been widely documented in evolutionary studies and may arise from several processes, including incomplete lineage sorting, introgression, sex‐biased dispersal, or differences in *N*_E_ between mitochondrial and nuclear genomes (Funk and Omland 2003; Toews and Brelsford 2012). MtDNA often exhibits stronger phylogeographic structure because it is maternally inherited, haploid, and has a smaller *N*_E_, which can lead to faster lineage sorting compared with nuclear markers (Avise 2012). In contrast, nuclear microsatellite loci reflect biparentally inherited variation and may capture more recent or ongoing gene flow among populations (Selkoe and Toonen 2006). Therefore, although the mitochondrial data suggest pronounced phylogeographic structuring and possible cryptic diversity within *Z. koreanus*, the contrasting patterns observed in nuclear markers indicate that these results should be interpreted cautiously. It would therefore be necessary to re‐evaluate the taxonomic status of *Z. koreanus* in the Nakdong and Seomjin River basins through integrative studies incorporating genomic, morphological, and ecological data. Similarly, according to Jeon et al. (2020), the Tamjin River population of the spined loach *Cobitis nalbanti* evolved a genetically divergent unique lineage, suggesting the possible presence of a cryptic species. Additional morphological and ecological analyses (e.g., geometric morphometrics on body shape/form) will further clarify species boundaries and potential hybrid forms.

Although the Han River and its tributaries were reported to be often geographically well‐separated (Chae and Yoon 2006), in this study, *Z. koreanus* populations from the Han River and East‐flowing rivers formed a monophyletic lineage (Figure 2). Unexpectedly, we found that clearly diversified and well distinguished clades of *Z. koreanus* individuals (Han River/East‐flowing river, Geum River, Nakdong River) co‐occurred between the respective river basins. The observed sharing of haplotypes could be most simply explained by artificial translocation by humans, given the considerable levels of genetic divergences between the clades (Figure 4). Several freshwater fish species (e.g., Korean splendid dace *Coreoleuciscus splendidus* and Korean torrent catfish *Liobagrus andersoni* ) occurred more frequently in the East‐flowing rivers, perhaps due to the increase of fish resources and indiscriminate introduction for fishery restorations. The number of *Z. koreanus* individuals has also increased rapidly compared to the past in East‐flowing rivers (Byeon and Oh 2015). Many cases of indiscriminate artificial introduction of freshwater fishes have been reported, often due to a direct release, as well as an indirect cause, in which multiple species were introduced in a mass release of fry during restoration action (Choi and Choi 2005; Park et al. 2013). In this sense, it would be conceivable that the *Z. koreanus* lineage of the Han River basin has been accidentally introduced to the East‐flowing rivers during ecological restoration activities. Also, *Z. koreanus* was originally distributed throughout most rivers in the Korean Peninsula, except for certain areas of the northeastern part of South Korea (e.g., Yeongdong province near the East‐flowing rivers), and has been translocated to that area, which may result in mixed clade distributions across river basins (Yoon et al. 2018). The observed genetic mixtures among *Z. koreanus* populations across distinct watersheds may be attributed not only to anthropogenic translocations but also to historical hydrogeomorphological processes, such as past river connections or river capture events. Freshwater fishes on both sides of the Taebaek Mountains (which is the headwaters of the Han and Nakdong Rivers) shared genetic similarities due to Quaternary hydrological connections (Kim et al. 2017). Given that major rivers such as the Han and Nakdong originate near the Taebaek region, past drainage connectivity may have naturally facilitated gene flow between populations (Kwan et al. 2018; Jeon et al. 2022).

### 4.2 Genetic Diversity and Structure of *Z. koreanus* Among/Within River Basins

*Zacco koreanus* populations had high haplotype diversity and low nucleotide diversity among haplotypes (*H* = 0.96, *π* = 0.014), which is consistent with those of closely related *Z. platypus* [*cyt b*; *H* = 1.00~0.74, *π* = 0.000~0.008 (Perdices et al. 2004)] and also those of other cyprinid species [naked carp *Gymnocypris przewalskii* ; COI and CR; *H* = 0.55, 0.93, *π* = 0.001, 0.006; (Fang et al. 2022)]. We found that the Han River populations show considerably higher levels of diversity than the other river basins (Table 1). The level of genetic diversity is known to be correlated with population sizes (Frankham 1996), and thus the Han River may house a much larger number of *Z. koreanus* individuals than the other rivers (Ministry of Environment 2019). While *Z. koreanus* is distributed widely in most streams in Korea, its major distributional areas are centered in the northern and central parts of South Korea (Kim et al. 2005). Consistent with this distribution pattern, higher genetic diversity was observed in the Han River populations. Moreover, *Z. koreanus* prefers habitats with low water temperatures and is therefore considered to be mainly distributed in the Han River, which has lower water temperatures than other river basins (Chae and Yoon 2006; Choi et al. 2011).

The finding of strong interriverine population structure and genetic divergence in *Z. koreanus* is most likely caused by limited gene flow among the geographically disconnected rivers, as those function as a physical barrier to dispersal (Baek et al. 2018; Kim, Jang, et al. 2020; Choi and Lee 2025). Natural populations of two freshwater species, Korean rosy bitterling *Rhodeus notatus* and short ninespine stickleback *Pungitius kaibarae*, in South Korea were also found to be strongly divergent in genetic structure among river basins, implying that the landscape isolated by each river drainage acts as a barrier to gene flow (Bae and Suk 2015; Jeon et al. 2020; Won et al. 2020). We found that even populations inhabiting geographically close areas (approximately 6 km apart) can be genetically distinct if they are geographically isolated. For example, the BDHR, SYNR, and BSGR populations belonging to the Han, Nakdong, and Geum Rivers, respectively, are located approximately 6 ± 1 km apart. Nevertheless, each population exhibited a distinct genetic lineage corresponding to a unique mtDNA clade (Clade 1, Clade 2, and Clade 3) specific to its respective river basins. Similarly, annual killifish *Nothobranchius* populations of Africa were genetically well‐separated, despite being located only a few kilometers apart, with rivers serving as an important barrier to gene flow and thus there was but a weak tendency for isolation by distance (Bartáková et al. 2015). Because fish can move within the same river basins, the connectivity of the river basins is more important for gene flow than the geographical distance per se (Underwood et al. 2016). *Z. temminckii* shows the same genetic cluster as the NR and SR populations of *Z. koreanus*, which suggests that interaction (genetic exchange) between the two species may be probable because the Nakdong and Seomjin Rivers are the only ecosystems where both species occur together (Kim, Han, et al. 2020).

However, the clustering pattern observed in the microsatellite analysis should be interpreted with some caution. The resolution of population genetic structure inferred from microsatellite data can be influenced by several factors, including the number of loci analyzed, levels of allelic diversity, and the transferability of markers developed from a limited number of populations (Selkoe and Toonen 2006; Barbará et al. 2007; Putman and Carbone 2014). In particular, analyses based on relatively few loci may sometimes have limited power to detect weak genetic differentiation among populations (Wang et al. 2021). Therefore, it is possible that the microsatellite markers used in this study may not fully capture fine‐scale genetic structure between *Z. koreanus* and *Z. temminckii* . Future studies incorporating a larger number of nuclear markers or genome‐wide datasets would help to clarify the extent of genetic differentiation and potential gene flow between these species. Similarly, nuclear markers such as RAG1/RAG2, S7 ribosomal protein intron, and Tmo‐4C4 have been widely used in teleost phylogenetic and population genetic studies because they represent independent biparentally inherited loci that can provide complementary evidence to mitochondrial datasets (Bufalino and Mayden 2010; Lin and Hastings 2013). Analyses based on multiple nuclear markers or genome‐wide datasets can help resolve mito–nuclear discordance and provide more robust inference of evolutionary relationships and population divergence (Hughes et al. 2018; Berbel‐Filho et al. 2022). Therefore, future studies incorporating these nuclear loci would help clarify whether the mitochondrial lineage divergence observed in *Z. koreanus* reflects historical isolation, ongoing gene flow, or early stages of lineage divergence.

### 4.3 Conservation Implications

The phylogeographic structure identified in this study may also have implications for the conservation of *Z. koreanus*. The mitochondrial analyses revealed geographically structured lineages largely corresponding to major river basins of the Korean Peninsula. Such lineage divergence among geographically isolated river basins may represent important components of genetic diversity within the species. From a conservation perspective, maintaining genetic diversity is widely recognized as an important factor for preserving evolutionary potential and long‐term population persistence (DeWoody et al. 2021). In addition, populations that are genetically differentiated across river basins may represent distinct conservation units because they may contain unique evolutionary or adaptive variation (Fraser and Bernatchez 2001). Also, *Z. koreanus* has often been used as an indicator species for assessing stream environmental conditions in Korea (Lee et al. 2013). However, the presence of cryptic genetic diversity or lineage divergence may have implications for its use in ecological assessments and biomonitoring programs (Bickford et al. 2007). Cryptic lineages within species can exhibit differences in ecological tolerance, life history traits, or environmental responses, which may influence the interpretation of ecological indicators (Feckler et al. 2014). Previous studies have highlighted that unrecognized cryptic diversity may bias ecological assessments and ecotoxicological evaluations if genetically distinct lineages are treated as a single species (Jourdan et al. 2023). Therefore, conserving populations across multiple rivers and recognizing genetic structure within species may help maintain evolutionary diversity, improve the reliability of biomonitoring, and support more effective freshwater biodiversity management.

Taken together, the results of this study show that *Z. koreanus* from South Korea has evolved four distinct mtDNA lineages in respective river basins and significant genetic structure among the river basins. In particular, the Nakdong and Seomjin River populations exhibit relatively high levels of genetic divergence, suggesting long‐term regional isolation and population‐level differentiation within *Z. koreanus*. However, mixed distributions of unique lineages were detected within *Z. koreanus*, suggesting artificial translocation by humans. This is the first study that provides important insights into the genetic structure of *Z. koreanus* populations from the major river basins in South Korea and also associates the results with ecological and evolutionary implications. The findings highlight a need for re‐evaluation of the taxonomic status of *Z. koreanus* in the Nakdong and Seomjin Rivers in relation to a close relative, *Z. temminckii* .

## Author Contributions

**Yu Rim Kim:** data curation (lead), formal analysis (lead), writing – original draft (lead). **Ji Eun Jang:** data curation (supporting), writing – review and editing (equal). **Hee‐kyu Choi:** investigation (lead), writing – review and editing (equal). **Soon Young Hwang:** formal analysis (supporting), writing – review and editing (equal). **Ji Young Kim:** conceptualization (equal). **Soonku So:** formal analysis (supporting), writing – review and editing (equal). **Hyuk Je Lee:** conceptualization (equal), formal analysis (supporting), funding acquisition (lead), supervision (lead), writing – original draft (lead).

## Funding

This research was supported by the Basic Science Research Program through the National Research Foundation of Korea (NRF) funded by the Ministry of Education (RS‐2024‐00412091), a NRF grant funded by the Korean government (RS‐2020‐NR054744), and also by a grant from the Korea National Park Research Institute (NPRI 2022‐05), Korea National Park Service and partly by Graduate School of the Sangji University.

## Conflicts of Interest

The authors declare no conflicts of interest.

## Supporting information


**Figure S1:** Estimation of the most likely number of genetic clusters (Δ*K*) based on the Delta *K* method of (Earl and Vonholdt 2012). Delta *K* values are plotted for *K* = 1–26. The highest peak observed at *K* = 2 (red vertical line) indicates that *K* = 2 is the optimal number of clusters for the analyzed populations.
**Figure S2:** STRUCTURE bar plots illustrating hierarchical genetic structure of *Zacco koreanus* (HR, HE, GR, NR, SR) and *Z. temminckii* (Zt) based on microsatellite data. Results are shown for Δ*K* = 3–6 and Δ*K* = 21, the latter corresponding to the second highest Δ*K* value. Each vertical bar represents an individual, and colors indicate proportional assignment to inferred genetic clusters.

## Data Availability

The mtDNA haplotype sequences and three mitogenomes obtained for this study have been deposited in GenBank under the accession numbers PV340652–PV340773.
