# Supplementary material for: Cryptic Diversity and Genetic Variation in a Korean Endemic Freshwater Fish, the Korean Dark Chub (Zacco koreanus) Compared With Its Relative Zacco temminckii
Source: Ecol Evol. 2026 Apr 8;16(4):e73451. doi: 10.1002/ece3.73451 (PMC13058722; doi:10.1002/ece3.73451)
Supplement: Supplementary file 1 — Figure S1: Estimation of the most likely number of genetic clusters (ΔK) based on the Delta K method of (Earl and Vonholdt 2012). Delta K values are plotted for K = 1–26. The highest peak observed at K = 2 (red vertical line) indicates that K = 2 is the optimal number of clusters for the analyzed populations. Figure S2: STRUCTURE bar plots illustrating hierarchical genetic structure of Zacco koreanus (HR, HE, GR, NR, SR) and Z. temminckii (Zt) based on microsatellite data. Results are shown for ΔK = 3–6 and ΔK = 21, the latter corresponding to the second highest ΔK value. Each vertical bar represents an individual, and colors indicate proportional assignment to inferred genetic clusters. [file ECE3-16-e73451-s001.docx]

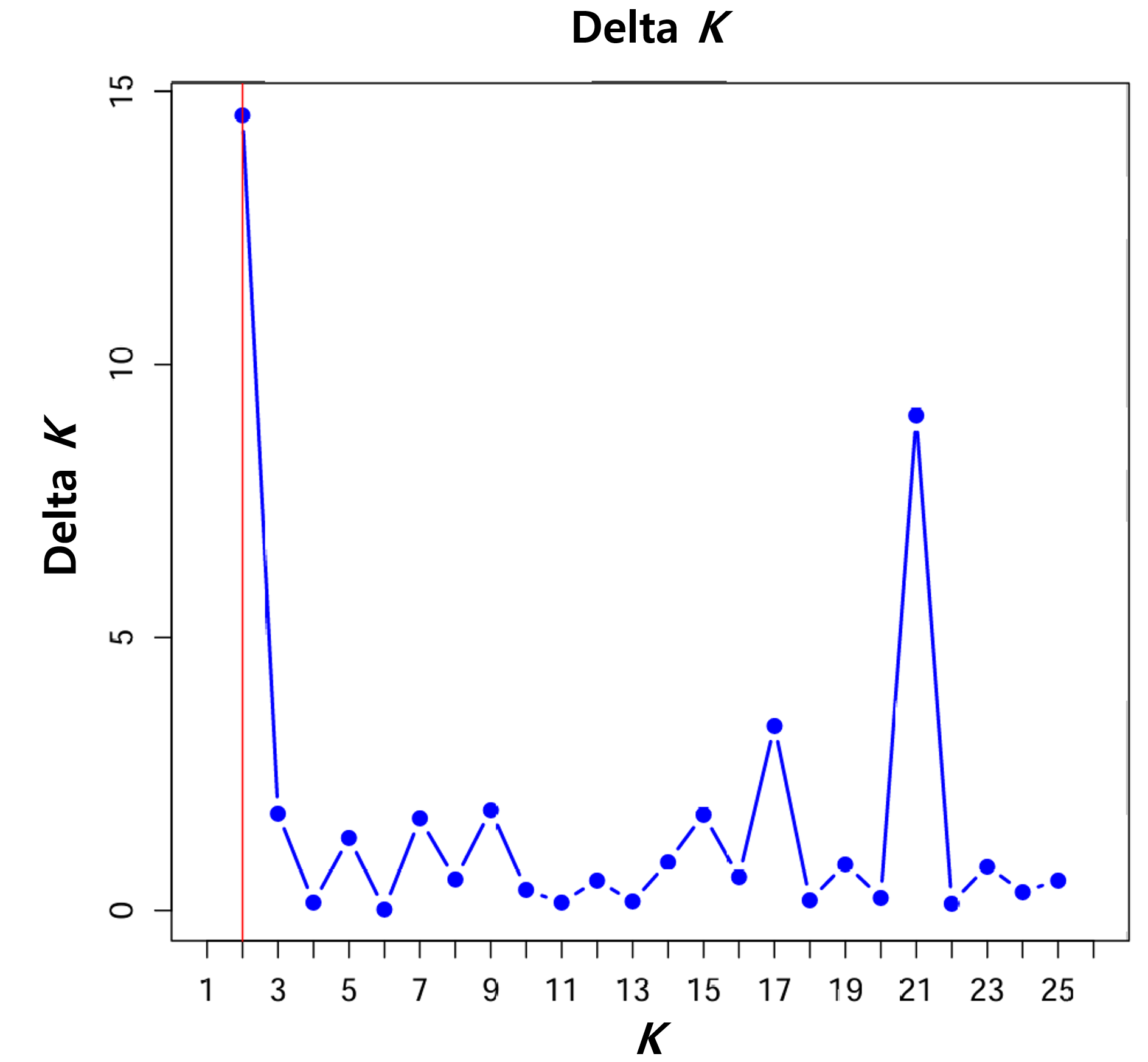


Figure S1. Estimation of the most likely number of genetic clusters (Δ*K*) based on the Delta *K* method of (Earl and VonHoldt, 2012). Delta *K* values are plotted for *K* = 1–26. The highest peak observed at *K* = 2 (red vertical line) indicates that *K* = 2 is the optimal number of clusters for the analyzed populations.

**
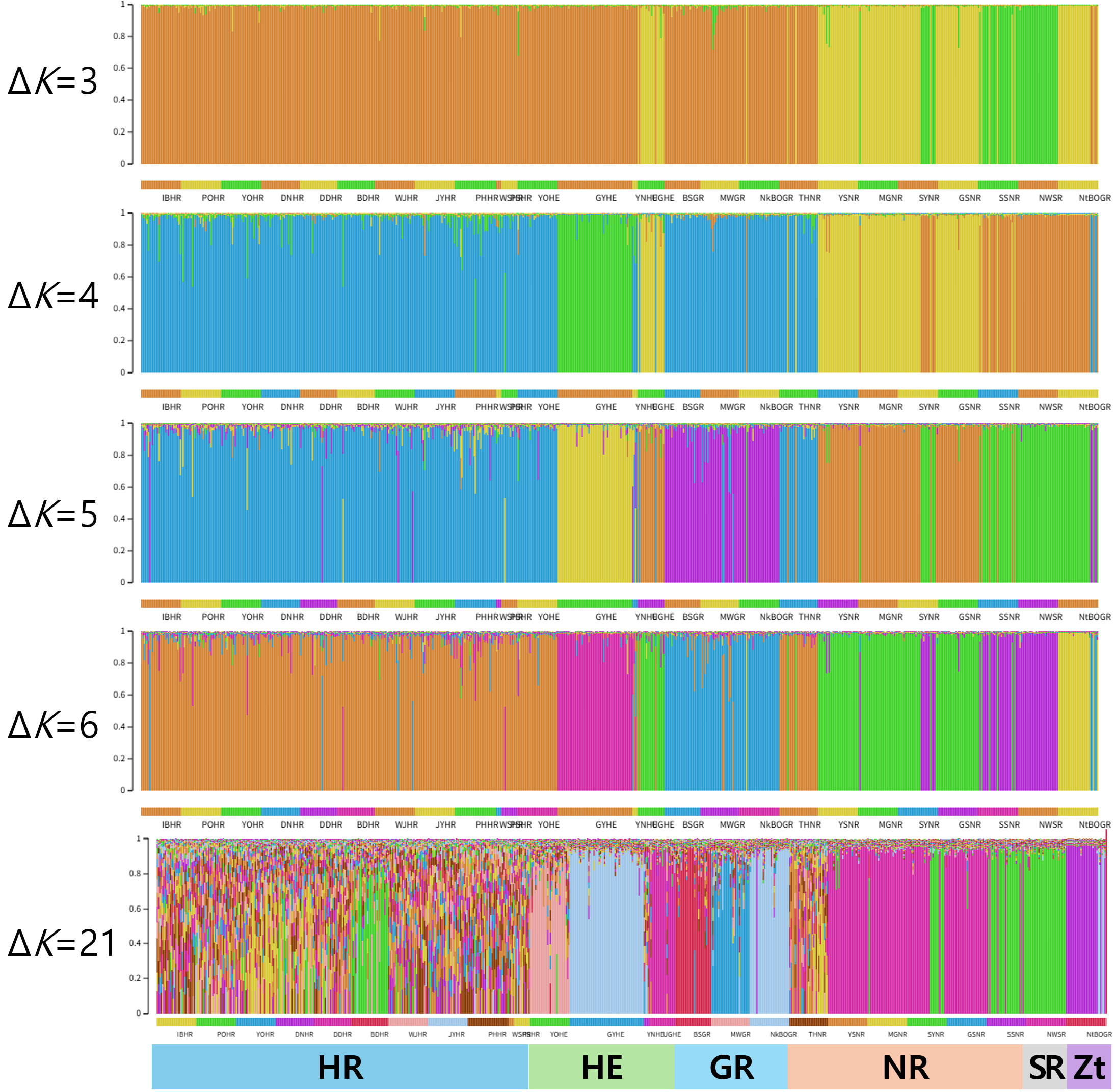
**

Figure S2. STRUCTURE bar plots illustrating hierarchical genetic structure of *Zacco koreanus* (HR, HE, GR, NR, SR) and *Z. temminckii* (Zt) based on microsatellite data. Results are shown for Δ*K* = 3-6 and Δ*K* = 21, the latter corresponding to the second highest Δ*K* value. Each vertical bar represents an individual, and colors indicate proportional assignment to inferred genetic clusters.
